# Model-Based Analysis of Impact, Costs, and Cost-effectiveness of Tuberculosis Outbreak Investigations, United States

**DOI:** 10.3201/eid3103.240633

**Published:** 2025-03

**Authors:** Sourya Shrestha, Lucia Cilloni, Garrett R. Beeler Asay, J. Steve Kammerer, Kala Raz, Tambi Shaw, Martin Cilnis, Jonathan Wortham, Suzanne M. Marks, David Dowdy

**Affiliations:** Johns Hopkins Bloomberg School of Public Health, Baltimore, Maryland, USA (S. Shrestha, L. Cilloni, D. Dowdy); Centers for Disease Control and Prevention, Atlanta, Georgia, USA (G.R.B. Asay, J.S. Kammerer, K. Raz, J. Wortham, S.M. Marks); California Department of Public Health, Richmond, California, USA (T. Shaw, M. Cilnis)

**Keywords:** tuberculosis and other mycobacteria, bacteria, TB outbreak investigation, TB modeling, cost-effectiveness analysis, United States

## Abstract

Outbreak investigation is an essential component of tuberculosis (TB) control in the United States, but its epidemiologic impact and cost-effectiveness have not been quantified. We modeled outbreak investigation activities in the United States during 2023–2032 and estimated corresponding epidemiologic impact, economic costs (in 2022 US$), and incremental cost-effectiveness ratios from the healthcare system perspective (cost per additional quality-adjusted life-year gained). We projected that outbreak investigations would result in 1,030,000 (95% uncertainty interval [UI] 376,000–1,740,000) contacts investigated, leading to 4,130 (95% UI 1,420–7,640) TB diagnoses and 104,000 (95% UI 37,600–181,000) latent TB infection diagnoses, at a total cost of US $219 million (95% UI $80–$387 million). We estimated that 5,560 (95% UI 1,720–11,400) TB cases would be averted through early detection and treatment, and the incremental cost-effectiveness of outbreak investigations, compared with no outbreak investigations, was $27,800 per quality-adjusted life-year gained (95% UI $4,580–$68,700).

Outbreak investigation continues to be an essential part of tuberculosis (TB) control in the United States (*1*–*3*). By promptly detecting and treating TB disease and latent TB infection (LTBI) among contacts of persons with TB or in settings in which TB transmission is likely to be ongoing, outbreak investigations play a critical role in curbing ongoing community transmission. TB incidence in the United States fell by >70% during 1993–2019, and widespread programmatic implementation of contact and outbreak investigations was likely a key contributor to this observed decline (*4*,*5*).

However, TB outbreaks continue to cause substantial illness, particularly in vulnerable populations, which include persons in racial and ethnic minority groups, persons living in congregate settings (such as correctional facilities and homeless shelters), and persons with underlying conditions, who have a higher predisposition to poor TB outcomes (*6*–*9*). Even though a minority of new TB cases (≈14%) in the United States are attributed to recent transmission (*10*,*11*), extensive public health resources are required for TB investigation and control, and outbreak investigations can present substantial financial and workload burdens to frontline public health departments. As such, outbreak prevention and control remain essential to eliminate TB in the United States, and improving the impact of these activities can further accelerate progress toward TB elimination goals. In this model-based analysis, we sought to estimate the epidemiologic effects of control efforts and quantify the cost-effectiveness of TB outbreak response efforts in the United States.

## Methods

### Projection of TB Outbreaks in the United States

We projected the number of TB cases, the number of TB clusters, and the distribution of cluster sizes in the United States during 2023–2032. TB cases were projected by extrapolating the trend of TB cases during 2014–2019 (1.1% annual decline) (*1*). To allow for additional uncertainty in TB incidence caused by other factors, such as the COVID-19 pandemic–related disruptions, we assumed that, relative to the prepandemic TB trajectory, from a 5% increase to a 10% decline could be seen in the number of cases projected during the study period (Tables 1, 2). We simulated cluster size distributions of TB outbreaks using a branching process model with a Poisson lognormal distribution (*12*). This model was previously developed and fitted to genotype cluster size distribution data in the United States (*12*), where cases were defined as clustered if they had matching spacer oligonucleotide typing (spoligotype) and 24-locus mycobacterial interspersed repetitive unit–variable number tandem-repeat genotyping results, and they were reported within the same state during 2012–2016 (*29*) (Appendix). Finally, we estimated the number of clusters on the basis of both the projected number of incident cases and the cluster size distribution, such that the sum of cases across simulated clusters equaled the projected number of incident cases.

**Table 1:** Descriptions, estimates and uncertainty ranges for parameters describing TB outbreaks and outbreak investigations in study of impact, costs, and cost-effectiveness of TB outbreak investigations, United States*

Model parameters	Point estimate	Lower value	Upper value	Sources and additional notes
Projection of TB cases and outbreaks				
Projected decline in TB cases, year-on-year % decline	1.06%	0%	2%	Based on year-on-year % decline in TB cases in the United States, 2014–2019 (*1*).
Change in TB incidence from the projected baseline because of other factors (e.g., COVID-19 pandemic)	No change	10% decrease	5% increase	Assumption. If is the annual rate of decline in TB cases before the pandemic, and is the impact of the pandemic, then the number of TB cases projected in the year is given by: .
R_0_	0.29	0.19	0.38	Shrestha et al. (*12*)†
Individual level heterogeneity, SD of the Poisson lognormal model	1.9	1.8	2	Shrestha et al. (*12*)†
Characterization of outbreak investigation				
Outbreak investigation threshold	>3 cases			Assumption, as in Mindra et al. (*2*).
No. contacts investigated per case during outbreak investigation	55	10	78	Mitruka et al. (*3*) reported 42 total contacts investigated per case among 27 outbreaks during 2002–2008; Mindra et al. (*2*) reported 88 contacts per case among 21 outbreaks during 2009–2015. We assumed that on average 10 contacts would be evaluated per case outside of outbreak investigation, on the basis of ARPE report (*13*), and that 5% of the case investigations occur as a part of outbreak investigation (Appendix).
% Contacts evaluated	79%	75%	85%	ARPE report (*13*)
% LTBI diagnoses in evaluated contacts	13%	10%	15%	Mitruka et al. (*3*), ARPE report (*13*)
% Contacts with LTBI initiating LTBI treatment	73%	70%	75%	ARPE report (*13*)
% Contacts with LTBI completing LTBI treatment	57%	55%	65%	ARPE report (*13*); this is a product of the percentage of contacts with LTBI initiating treatment, and percentage of those initiating that complete treatment.
% Evaluated contacts with TB disease	0.5%	0.29%	0.72%	Mitruka et al. (*3*) reports 0.62%; 0.72% by ARPE report (*13*); Mindra et al. (*2*) reports 0.29%.

*3HP, 3 months of isoniazid and rifapentine; 9H, 9 months of isoniazid; ARPE, Aggregate Reports for Program Evaluation; CDC, Centers for Disease Control and Prevention; IGRA, interferon-γ release assay; LTBI, latent TB infection; QALY, quality-adjusted life-years; R_0_, basic reproduction number; TB, tuberculosis. †Based on Poisson lognormal distributions fitted to cluster-size distribution of genotype linked cases in the United States during 2012–2016.

**Table 2:** Descriptions, estimates and uncertainty ranges for parameters describing TB natural history, costs of TB outbreak investigations, and cost-effectiveness evaluation in study of impact, costs, and cost-effectiveness of TB outbreak investigations, United States*

Model parameters	Point estimate	Lower value	Upper value	Sources and additional notes
Characterization of TB natural history and the impact of intervention				
% Contacts who will develop TB within 5 years after infection	6.6%	3%	15%	Based on estimates of reactivation of LTBI among recent exposure (*14*) and among close contacts of TB patients (*15*). Lower value of 3% reflects uncertainty in the recency of the infection among contacts.
Efficacy of completed LTBI treatment	93%	70%	95%	Estimates from 9H trial and noninferiority of 3HP compared with 9H (*16*,*17*).
R_0_ of cases detected during outbreak investigation	0.29	0.15	1.5	R_0_ of 0.29 from Shrestha et al. (*12*). Upper value of 1.5 for R_0 _reflects outbreak settings with higher transmission.
% Reduction in infectious period through early detection	50%	25%	75%	Modeled as reduction in R_0_ based on higher case detection and notification in contact investigations (*18*), resulting in reduction in delays in TB diagnosis, a contributor to outbreaks (*2*).
Unit cost estimates, 2022 US$				
Cost of PCR-based genotyping, per isolate	$35	$25	$50	CDC (culture, typing, and identification by nucleic acid probe, amplified probe technique) (*19*)
Cost of outbreak investigation, cost per contact during outbreak investigation†	$151	$86	$225	Unpublished data from 2 outbreaks in California (average cost of $106 per contact (2014 US$) (T. Shaw, unpub. data) (Appendix); unpublished CDC data reports mean cost of $175.90 ($78.00–$293.50) (2022 US$) (*20*).
Cost of LTBI testing per contact	$71	$60	$80	Includes costs of IGRA LTBI testing, and costs of chest radiograph and TB test to rule out TB disease among those testing positive for IGRA (*19*,*21*,*22*).
Cost of LTBI treatment per infected contact	$515	$300	$700	Includes costs of 3HP (*23*), laboratory testing, and toxicity both requiring and not requiring hospitalization (*24*). Assumes toxicity among 3.2% (*20*) of persons receiving LTBI treatment (*25*), and 0.015% requiring hospitalization (*26*).
Cost of TB treatment per contact with disease	$23,543	$15,000	$30,000	Direct TB treatment costs for non–MDR TB (*27*)
QALY estimates				
Annual discount rate	3%			Assumption
QALYs gained per TB case averted	1.16	0.74	1.39	Assumes 4.7% average mortality among people with TB, 36.3 years of average life expectancy at TB diagnosis, and health utility of 0.76 during TB treatment (*28*).‡
QALYs lost per LTBI treatment	0.002	0.0015	0.0025	Jo et al. (*20*).§

*3HP, 3 months of isoniazid and rifapentine; 9H, 9 months of isoniazid; ARPE, Aggregate Reports for Program Evaluation; CDC, Centers for Disease Control and Prevention; IGRA, interferon-gamma release assay; LTBI, latent TB infection; MDR, multidrug resistant; QALY, quality-adjusted life-years; TB, tuberculosis. †Excludes costs associated with TB and LTBI treatment. ‡See Appendix for details on TB mortality rates; other data were incorporated to construct the range. §QALY estimate assumes toxicity among 3.2% of persons receiving LTBI treatment (*25*), and 0.015% requiring hospitalization (26).

### Epidemiologic Impact of Outbreak Investigation

For the purposes of this analysis, we assumed that all genotype clusters of >3 cases would be considered for outbreak investigation response, consistent with assumptions in previous outbreak investigation reviews (*2*). We estimated the number of contacts investigated per case during an outbreak investigation using historical data on outbreak investigations in the United States (*2*,*3*), accounting for contact investigations expected to occur outside of outbreak investigation based on the Aggregate Reports for Program Evaluation (ARPE) from reporting jurisdictions (50 states and 9 cities) to the Centers for Disease Control and Prevention (CDC) (*13*) (Appendix). Among the contacts investigated during the outbreak response, we estimated the number of persons who would be identified as having TB disease and LTBI on the basis of historical data on outbreak investigations and from contact investigations (*2*,*3*,*13*). For persons who tested positive for LTBI, we also estimated the proportion who would initiate and complete LTBI treatment on the basis of data reported in ARPE (*13*).

We assumed that outbreak investigations would result in earlier detection of TB, thus also preventing further transmission by reducing the number of secondary cases by 50% (with sensitivity analysis including an uncertainty range of 25%–75%). On the basis of historical data (*2*,*3*,*13*), we assumed that TB disease would be diagnosed and treatment would be initiated in 0.5% (uncertainty range 0.29%–0.72%) of contacts; we assumed that LTBI would be diagnosed in 13% (uncertainty range 10%–15%) of contacts, and 57% (uncertainty range 55%–65%) of them would initiate and complete LTBI treatment (*13*) with an efficacy of 93% (uncertainty range 70%–95%) (*16*,*17*). We assumed that 6.6% (uncertainty range 3%–15%) of persons in whom LTBI was diagnosed during an outbreak investigation would develop reactivation TB within 5 years (in the absence of LTBI treatment), on the basis of published estimates of progression after recent exposure or progression specifically among close contacts (*14*,*15*).

### Cost-effectiveness of Outbreak Investigation

We used a TB-centered health systems perspective to estimate costs and cost-effectiveness and focused on incorporating costs and benefits that are directly related to TB-related services and outcomes. We relied on published literature and other sources to estimate costs associated with outbreak investigations (Tables 1, 2). We incorporated costs of genotyping, assuming all cases (including those that might end up being part of outbreak investigation) are genotyped; conducting outbreak investigation on all outbreaks of >3 cases (excluding TB and LTBI treatment costs); and testing contacts of outbreak TB cases for LTBI and treating persons who tested positive. Unit costs of genotyping included the costs of culturing *Mycobacterium tuberculosis*, genotyping the isolate, and nucleic acid amplification at the CDC laboratory. Outbreak investigation costs were based on 2 sources. The first source was unpublished data from 2 outbreaks in northern California during 2010–2014 (T. Shaw, unpub. data). A total of 276 contacts were investigated across the 2 outbreaks; the corresponding total cost was $29,238 ($106 [in 2014 US dollars] per person investigated), including coordination and communication, analytical activities, case management, contact identification, and evaluation (Appendix). We also used CDC data from Njie et al. (*20*), who reviewed the costs of contact investigations across the United States and estimated the mean cost to be $175.90 (95% CI $78.00–$293.50) per contact. In estimating unit costs of testing and treatment, we assumed that interferon-gamma release assays were used for LTBI testing and that treatment consisted of the 3HP regimen (3 months of self-administered isoniazid and rifapentine) (*21*). We accounted for the costs of chest radiography (*19*,*22*) and laboratory costs (*23*), as well as toxicity and hospitalization during treatment (*24*–*26*). The averted future costs of TB treatment likewise included inpatient and hospitalization costs (*30*,*31*) (Tables 1, 2). We assumed that outbreak investigation costs were distributed evenly over the 10-year analytic period, that future cases resulting from exposure during outbreaks occurred within 5 years of completing the outbreak investigation, and that those cases were distributed exponentially over the 5-year period. We measured all costs from the health system perspective and matched costs to 2022 dollars using the Health Care Price Index for personal consumption expenditures from the US Bureau of Economic Analysis (*32*). We discounted future costs and cost savings at 3% annually.

The primary cost-effectiveness outcome was the incremental cost-effectiveness ratio (ICER) of outbreak investigation activities (cost per quality-adjusted life year [QALY] gained) during 2023–2032, comparing a baseline scenario in which outbreak investigations are conducted (using conventional genotyping) to a counterfactual scenario in which no outbreak investigation activities are conducted. Following the approach taken by Jo et al. (*21*), we estimated the net number of QALYs gained as the difference between the total QALYs gained, resulting from averted future TB cases and averted disabilities among those that are diagnosed during outbreak investigations, and QALYs lost because of the toxicity of treatment. QALY losses associated with TB disease included TB-related mortality rates (*23*) and loss of quality of life during TB treatment (*28*) and QALY losses associated with LTBI treatment, including both toxicity (*25*) and hospitalization (*26*) during treatment (Appendix). 

### Model Simulation

We used a Monte Carlo approach to generate estimates of our model outcomes. We performed 10,000 model simulations, each using a parameter set generated by probabilistically sampling model parameters. Each model parameter was drawn from a triangular distribution, where the mode of the distribution was taken to be the point estimate, and the range of the distribution varied between the lower and the upper values (Tables 1, 2). For each outcome, we reported the mean and the 95% uncertainty interval (UI; 2.5th–97.5th percentiles) across all model simulations. As a sensitivity analysis of our choice of parameter distribution, we also considered PERT (program evaluation and review technique) distribution and a mixture of PERT and gamma distributions (Appendix). We performed model simulations by using R software (The R Project for Statistical Computing, https://www.r-project.org). 

### Sensitivity Analyses

We conducted a multivariate sensitivity analysis to explore the sensitivity of the primary outcome to uncertainty in parameter values. We varied all model parameters across specified ranges according to parameter-specific distributional assumptions and compared the difference in projected incremental cost-effectiveness between the 1,000 simulations in which the value of the parameter of interest was in the top decile and the 1,000 simulations in which that value was in the bottom decile.

## Results

**Figure 1:**
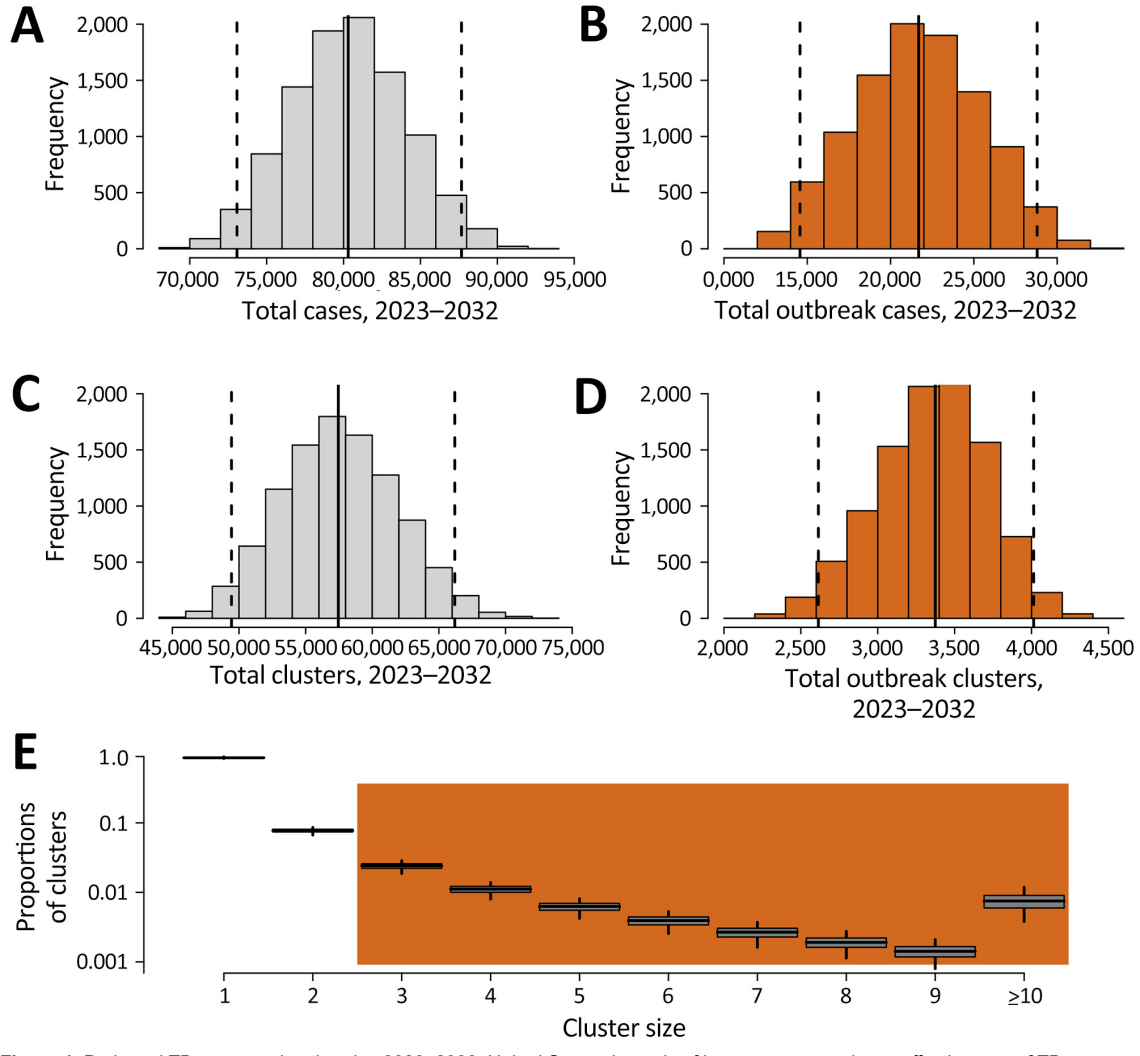
Projected TB cases and outbreaks, 2023–2032, United States, in study of impact, costs, and cost-effectiveness of TB outbreak investigations. Shown are the total number of TB cases (A), the number of TB cases occurring in outbreaks (B), the total number of TB clusters (C), and the number of TB outbreaks (i.e., clusters of >3 cases) (D) projected to occur during 2023–2032 and cluster size distributions of the TB clusters (E). Clusters of size 1 are assumed to have no transmission links, and only clusters with >3 cases (shaded in orange) are investigated in outbreak investigations.

We projected that 3,350 (95% simulation UI 2,610–4,010) outbreaks (clusters with >3 TB cases) would occur in the United States over the 10-year period of 2023–2032 and that those outbreaks would include a total of 21,700 (95% UI 14,600–28,800) cases (Figure 1). During this period, we estimated that 1.03 million (95% UI 376,000–1,740,000) persons who had contact with an outbreak TB case would require investigations. Of those persons, we projected that TB disease would be diagnosed in 4,130 (95% UI 1,420–7,640) persons and LTBI would be diagnosed in 104,000 (95% UI 37,600–181,000) persons, reflecting recent infection during the outbreak. Of those persons, we estimated that 61,500 (95% UI 22,000–107,000) would successfully complete LTBI treatment (Table 3).

**Table 3:** Projected scope of TB outbreak investigations during 2023–2032 in study of impact, costs, and cost-effectiveness of TB outbreak investigations, United States*

Projected outcome	Point estimate	Lower bound	Upper bound
No. TB outbreaks with >3 cases investigated	3,350	2,610	4,010
No. outbreak-related TB cases	21,700	14,600	28,800
No. contacts investigated during outbreak investigations	1,030,000	376,000	1,740,000
No. TB cases detected	4,130	1,420	7,640
No. contacts with LTBI detected	104,000	37,600	181,000
No. contacts with LTBI initiating treatment	75,700	27,100	131,000
No. contacts with LTBI completing treatment	61,500	22,000	107,000

*The point estimate is the mean, and the lower and upper bounds represent the 2.5th and 97.5th percentiles across all 10,000 simulations. LTBI, latent TB infection; TB, tuberculosis.

We estimated that 1,330 (95% UI 266–3,650) cases of future TB would be averted by outbreak investigation through early detection and averted transmission and an additional 4,220 (95% UI 1,200–9,090) cases would be prevented by treating LTBI. Thus, a total of 5,560 (95% UI 1,720–11,400) projected future cases would be averted in the United States through outbreak investigation over the 10-year period of 2023–2032 (Table 4).

**Table 4:** Projected epidemiologic impact of TB outbreak investigations, United States, 2023–2032*

Projected outcome	Point estimate	Lower bound	Upper bound
No. TB cases averted from early detection†	1,330	266	3,650
No. TB cases averted through LTBI treatment‡	4,220	1,200	9,090
Total TB cases averted	5,560	1,720	11,400

*The point estimate is the mean, and the lower and upper bounds represent the 2.5th and 97.5th percentiles across all 10,000 simulations. LTBI, latent TB infection; TB, tuberculosis. †Includes cases expected from transmission that would have occurred within five years, in the absence of outbreak investigation. ‡Includes cases expected to result from reactivation of LTBI that would have occurred within five years among persons who are detected with LTBI and complete LTBI treatment during an outbreak investigation.

We estimated that outbreak investigation activities would cost a total of $219 million (95% UI $80–$387 million) during 2023–2032. This total cost includes the costs of genotyping (1% of the total cost), conducting outbreak investigations (62%), LTBI testing (22%), and LTBI treatment (15%). The estimated (discounted) cost of preventing TB that would be averted through outbreak investigation was $102 million (95% UI $29.7–$216 million), for a net cost of $109 million (95% UI $24.7–$249 million) over 10 years. We estimated the ICER of outbreak investigations compared with no outbreak investigations to be $27,800 per additional QALY gained (95% UI $4,580–$68,700) (Table 5). Our estimates of the epidemiologic effects and cost-effectiveness were robust to the choice of parameter distributions (Appendix).

**Table 5:** Cost and cost-effectiveness (in 2022 US dollars) of TB outbreak investigation, United States, 2023–2032*

Projected outcomes	Point estimate	Lower bound	Upper bound
Costs of genotyping, millions	$2.5	$1.85	$3.24
Costs of outbreak investigation, millions	$135.0	$46.6	$251.0
Costs of LTBI testing, millions	$49.0	$17.7	$84.4
Costs of LTBI treatment, millions	$32.4	$11.0	$60.6
Total cost, millions	$219.0	$80.0	$387.0
Averted future costs of TB treatment, millions†	($102.0)	($29.7)	($216.0)
QALYs gained by averted TB	4,890	1,490	103,000
QALYs gained by averted TB-related disabilities through early diagnosis	183	51.4	4020
Total QALYs gained	5,070	1,560	106,000
QALYs lost during LTBI treatment	98.7	34.6	177.0
Cost per QALY gained	$27,800	$4,580	$68,700

*The point estimate is the mean, and the lower and upper bounds represent the 2.5th and 97.5th percentiles across all 10,000 simulations. LTBI, latent TB infection; QLY, quality-adjusted life-years; TB, tuberculosis. †Future costs of treating future cases are discounted on the basis of their expected time of occurrence up to 5 years into the future.

**Figure 2:**
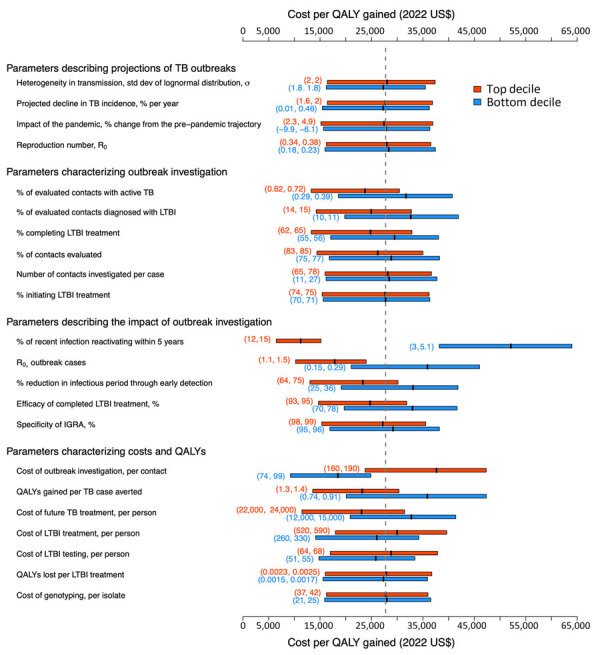
Multivariate sensitivity analysis of the model parameters in study of impact, costs, and cost-effectiveness of TB outbreak investigations, United States. This graph illustrates sensitivity of the incremental cost-effectiveness ratio of TB outbreak investigation in the United States (cost in 2022 US$ per QALY gained, compared with no outbreak investigation) to the values of individual model parameters. Each pair of boxplots shows variation in the outcome when the analysis was limited to either simulations in which the value of the parameter of interest was in the top (red) or bottom (blue) decile of its values across all simulations. The edges of each box represent the lower and upper interquartile range, and the band in the middle represents the mean. The vertical dashed line shows the mean across all simulations ($27,800 per QALY gained, corresponding to the primary outcome). The numbers within parentheses represent the parameter range (up to 2 significant figures) for the top (red) or the bottom (blue) decile. IGRA, interferon-γ release assay; LTBI, latent TB infection; QALY, quality-adjusted life-years; R_0_, basic reproduction number; TB, tuberculosis.

The factors that were most influential to the estimated cost-effectiveness of outbreak investigation activities consisted of characteristics of outbreak investigations, such as the proportion of contacts that were TB and LTBI cases (Figure 2); epidemiologic quantities, such as the reactivation rate of LTBI cases among outbreak contacts and the number of secondary transmissions per outbreak-related case; and cost-related variables, such as the cost of outbreak investigation (cost per contact) and the cost of treating future TB cases. Under all variations of parameter values evaluated in the sensitivity analysis, the estimated ICER did not exceed $70,000 per additional QALY gained.

## Discussion

In this model-based analysis of TB outbreak investigation in the United States during 2023–2032, we projected that outbreak investigation activities could prevent 5,560 cases of TB, a number equal to ≈6% of all incident cases (and ≈40% of all recent transmission cases) expected to occur in the country during that time (*5*,*10*,*11*). Furthermore, compared with other TB interventions in the United States, such as targeted testing and treatment of LTBI that has been previously evaluated as cost-effective in populations at risk for TB (*33*,*34*), outbreak investigation activities are generally more cost-effective at ≈$28,000 per QALY gained in the most likely scenario (<$70,000 per QALY gained in the most pessimistic scenarios). Those results strongly support maintaining TB outbreak response activities in the United States.

Historical data from outbreak investigations in the United States show that outbreak response activities are highly effective in finding both persons with TB disease and those with LTBI (*2*,*3*,*13*,*35*). For example, the prevalence of TB among persons investigated during outbreak investigations is >100 times the prevalence of TB in the general population, and the prevalence of LTBI are 3–5 times higher (*5*,*36*,*37*). Furthermore, early diagnosis and treatment of TB and LTBI during outbreak investigation is more likely to prevent future TB disease that would have occurred through transmission and reactivation. Factors that contribute to the occurrence of outbreaks, including higher prevalence of known risk factors such as substance use (*7*), barriers in access to care (*38*), and congregate living arrangements (*6*,*9*), also result in higher risks of ongoing transmission if TB is not promptly diagnosed and treated (*39*). In addition, persons in whom LTBI is diagnosed during outbreaks are more likely to have been exposed recently and, therefore, are at substantially higher risk for progression (*14*,*15*). Thus, even as the prevalence of TB in the United States continues to decline, robust outbreak response activities are likely to remain cost-effective.

This analysis assumed conventional PCR-based genotyping methods to define outbreaks. However, in 2018, the CDC began implementing whole-genome sequencing (WGS)–based genotyping methods; the costs associated with using those methods might differ from PCR-based genotyping and might also enable increased discriminatory power, as well as the ability to perform more detailed analyses and exclude genetically distant cases from the outbreak investigation (*40*,*41*). In addition, drug resistance testing can be performed with WGS, reducing the need for separate drug susceptibility testing. Finally, we exclusively modeled 3HP as the LTBI treatment regimen, but other regimens are also being used. Future analyses could refine our estimates by incorporating the costs and benefits of WGS for outbreak investigation and changes in LTBI treatment regimens.

TB outbreaks vary substantially in size and by setting, geography, and context (*12*). Because most outbreak investigations are conducted locally, the costs and extent of outbreak response activities also vary widely from one outbreak to the next and from one location to the next. We relied on historical data, such as those reported in reviews of outbreak investigations (*2*,*3*) and the ARPE (*14*), to estimate the average extent of TB outbreak response activities and used data from a small number of outbreak investigations to estimate corresponding costs. Those outbreak investigations might not be representative of the spectrum of outbreak investigations that are conducted across the country and across a variety of settings. Furthermore, the type of activities that constitute an outbreak investigation, including analytical activities, communication, and coordination, are likely to vary by setting and context. As such, our estimates should not be taken as reflective of any single outbreak investigation in any specified location but rather as an average estimate with substantial variability, as reflected in the uncertainty around our projections. More detailed data are required to better characterize variations in cost and cost-effectiveness of outbreak investigation across the United States, including how costs might scale with the size of the corresponding outbreak. Our projections allow for some uncertainty in the future trends in TB. However, some factors, such as changes in future immigration patterns and their effects, are harder to project.

Our analysis used a TB-centered health systems perspective and excluded patient costs, non-TB related healthcare costs, and other societal costs (e.g., reductions in productivity because of workplace closures during outbreaks or because of TB disease); taking a more comprehensive societal perspective might provide an even stronger rationale for investing in outbreak investigation (*42*). In addition, including potential averted disability resulting from post-TB sequelae might further improve the estimated cost-effectiveness of this intervention (*43*). Conversely, this analysis also did not include public health infrastructural costs that might be required to establish and maintain outbreak investigation and surveillance programs. Such costs could be substantial but are difficult to quantify at the national level. Finally, our approach does not consider the potential value of TB outbreak response activities from the perspective of improving equity (*44*). Given that TB outbreaks disproportionally affect persons in racial and ethnic minority groups and persons affected by poverty (*6*,*7*), TB outbreak investigation activities might also help reduce health disparities.

In conclusion, this model-based analysis predicts that TB outbreak response activities are likely to be both epidemiologically impactful and relatively cost-effective compared with other interventions in the United States over the next decade. A better understanding of the drivers of transmission in outbreaks, concerted efforts to document the scope and costs of outbreak response activities (especially by outbreak setting), and accounting for the use of novel tools such as WGS could further improve future estimates of the effects and cost-effectiveness of TB outbreak response.

Appendix Additional information about model-based analysis of impact, costs, and cost-effectiveness of tuberculosis outbreak investigations, United States
