# Supplementary material for: Model-Based Analysis of Impact, Costs, and Cost-effectiveness of Tuberculosis Outbreak Investigations, United States
Source: Emerg Infect Dis. 2025 Mar;31(3):497–506. doi: 10.3201/eid3103.240633 (PMC11878319; doi:10.3201/eid3103.240633)
Supplement: Appendix — Additional information about model-based analysis of impact, costs, and cost-effectiveness of tuberculosis outbreak investigations, United States [file 24-0633-Techapp-s1.pdf]

Article DOI: https://doi.org/10.3201/eid3103.240633

EID cannot ensure accessibility for supplementary materials supplied by authors. Readers who have difficulty accessing supplementary content should contact the authors for assistance.

Model-Based Analysis of Impact, Costs, and Cost-Effectiveness of Tuberculosis Outbreak Investigations, United States

Appendix

Summary of California TB outbreak investigations

As a part of the Tuberculosis Outbreak Prevention Feasibility Project, the California Department of Public Health investigated two outbreaks of tuberculosis (TB) during 2013–2015, one in San Mateo County and one in Alameda County. Across the two outbreaks, a total of 276 contacts were identified, of which 202 were evaluated. Three of the contacts were diagnosed with TB disease, and 31 with latent TB infection (LTBI). A total of 704.25 hours were spent on outbreak investigation activities, summarized in Appendix Table 1. A total of U.S.$ 29,238 was spent on these outbreak investigation activities, at an average cost of U.S.$106 per contact.

Appendix Table 1. Outbreak investigation activities during two TB outbreaks in California

Outbreak investigation activities	Hours spent	Percentage
Analytical Activities	103	15%
Case Management	62.5	9%
Contact Evaluation	64.25	9%
Contact Identification	33.5	5%
Contact Treatment	3.5	0%
Coordination and Communication	437.5	62%
Total	704.25	100%

TB case fatality ratios

Based on the TB case notification and mortality data (summarized in Appendix Table 2), we estimated the average age of individuals with TB attributed to recent transmission (RT-TB) to be 44.9 years (rounded to 45 years). We assumed that RT-TB cases were more representative of TB cases likely to occur in TB outbreaks. Based on the age-specific case-fatality ratios and the proportion of RT-TB cases occurring in each age group, we estimated the overall TB case-fatality ratio among RT-TB cases

to be 4.7%. Compared to all TB cases, RT-TB cases were relatively younger and had lower TB case fatality ratios.

Appendix Table 2. TB case fatality ratios

Age group	Age-specific TB case fatality ratios†	Percentage of Cases Attributed to Recent Transmission (RT)‡
0–4 y	0.006	2.9%
5–14 y	0.005	1.8%
15–24 y	0.006	12.3%
25–44 y	0.012	31.7%
45–64 y	0.049	36.8%
65 y and above	0.168	14.5%

†Based on TB cases and deaths among TB cases in the United States between 1999 and 2016.
‡Based on TB cases attributed to recent transmission (RT) in the United States between 2011 and 2019.

Details for QALY estimates

We estimated QALYs per TB case accounting for the loss in quality of life during TB disease and the QALYs associated with TB-related mortality.

Appendix Table 3. Parameters and assumptions for QALY estimates.

Parameter	Point Estimate	Lower value	Upper value	Notes/sources
Average age of an individual with TB disease occurring in an outbreak	45 y	40 y	50 y	Based on age-specific incidence of TB cases in the United States between 2011–2019, attributed to recent transmission, summarized on Appendix Table 2.
Remaining life expectancy, L	36.3 y	31.8 y	40.9 y	Based on 2019 U.S. life expectancy by age (*1*). Low value corresponds to 50 y and upper value corresponds to 40 y.
Quality of life (or health utility value) for individuals without TB, Q	1	0.8733	1	Lower value based on measurements of health-related quality of life among control participants (individuals without TB) in Montreal (*2*) and as adapted by Dale et al. (*3*) The upper value based on prior literature (*4*–*6*).
Annual discount rate, r		3%		Assumption
QALYs associated with TB fatality, Q_f	22.1	16.3	23.6	QALYs associated with TB fatality = $\frac{Q-e^{-rL}}{r}$, where Q is the quality of life without TB; L is the expected life remaining, and r is annual discount rate.
TB case fatality ratio, f	0.047	0.042	0.052	Based on estimated TB case fatality ratio among TB cases attributed to recent transmission, summarized in Appendix Table 2.
QALYs per TB case resulting from TB-associated mortality, $QALY_f$	1.04	0.68	1.32	$Q_f * f$ where Q_f is the QALY per fatal case, and *f* is the TB case fatality ratio.
Quality of life (or health utility value) for individuals with TB disease, Q^D	0.76	0.67	0.8182	Point estimate based on Guo et al. (*7*) Lower value based on Salomon et al. (*8*) Upper value based on annual utility values estimated by Dale et al assuming a 12 mo period of disease with various levels disabilities (*3*).
QALYs per TB case resulting from TB-associated non-fatal loss in quality of life, $QALY_{nf}$	0.12	0.05	0.16	QALYs resulting from TB-associated non-fatal loss in quality of life = $\frac{(Q-Q^D)(1-e^{-rD})}{r}$, where Q and Q^D are the quality of life without TB and with TB, respectively; *D* is the average time

Parameter	Point Estimate	Lower value	Upper value	Notes/sources
				with the disease, and r is annual discount rate. We assume a 6-mo period of TB disease for the point estimate and upper value, and a 12 mo period of disease, as in Dale et al. (*3*), for the upper value. Lower value is based on the lower value of Q and the upper value of Q^D; upper value is based on upper value of Q and the lower value of Q^D.
Total QALYs per TB case	1.16	0.74	1.39	$QALY_f + QALY_{nf}$

Estimating the number of contacts investigated per case

Mitruka et al. (*9*) reported that 42 total contacts were investigated per case among 27 outbreaks during 2002–2008, and Mindra et al. (*10*) reported 88 contacts per case among 21 outbreaks during 2009–2015. Taking the average between these two reports, we estimated that, on average 65 contacts were investigated per case. To account for investigations that would have happened as a part of routine contact investigation outside the context of an outbreak investigation, we first estimated the average number of contacts that are investigated per case in contact investigations, specifically in non-outbreak scenarios.

Based on the aggregated contact investigation data in the United States during 2015–2019 (*11*), across all forms of investigations, 13.2 contacts were investigated per case, on average. We estimated that ≈5% of the cases included would have occurred as a part of an outbreak (i.e., in transmission clusters of 3 or more cases, following the definition we have adopted in this analysis). If x contacts per case were evaluated for cases in non-outbreak scenarios, then the weighted average of contacts evaluated per case in an outbreak and non-outbreak scenarios would be equal to the reported average of 13.2 contacts per case, leading to the following equation:

$$(0.05) * 65 + (1 - 0.05) * x = 13.2$$

and $x \approx 10.47$.

Hence, we estimated that 55 out of the 65 (i.e., 65–10) contacts per case would have been investigated during outbreak investigation.

Outbreak Model

In this model, the number of secondary cases resulting from a single case is given by the "offspring distribution" of the branching process model, Z. This probability distribution follows a

Poisson distribution, i.e., $\boldsymbol{P}(Z = z) \sim \text{Poisson}(\nu)$, where the individual reproductive numbers ν follow a lognormal distribution (i.e., $\exp(\nu) \sim N(\mu, \sigma^2)$), in which μ and σ are the mean and standard deviation, respectively, of an underlying normal model. The mean of this distribution, the average number of secondary cases resulting from a single case, is the reproductive number, R_0. Standard deviation, σ, which characterize the heterogeneity in transmission, and the reproductive number, R_0, were previously fitted to genotype cluster size distribution in the United States between 2012–2016 (*12*). For the genotype clusters considered here, cases were defined as clustered if they (a) had matching spacer oligonucleotide typing (spoligotype) and 24-locus mycobacterial interspersed repetitive unit—variable number of tandem repeats (MIRU-VNTR) genotyping results, (b) were reported within the same state, and (c) occurred during 2012–2016 (*13*).

Analytic time horizon

We assume that the outbreak investigations are implemented over a 10-year period between 2023 and 2032, with the effort and costs equally distributed over the 10-year period (i.e., 10% of the overall effort and undiscounted costs occur each year during 2023–2032; See Appendix Figure 1). We consider the impact of the intervention (i.e., future TB cases averted) over a 5-year period post-intervention and assume that the impact of the intervention decreases exponentially over the time period, consistent with findings that most TB reactivations occur within 5 years post-infection (*14*) and that TB reactivation risk decrease almost exponentially after infection (*15*). We thus consider a 15-year time horizon, 2023–2037, to account for the potential overall impact of the intervention implemented during 2023–2032 (See Appendix Figure 1, dashed red line).

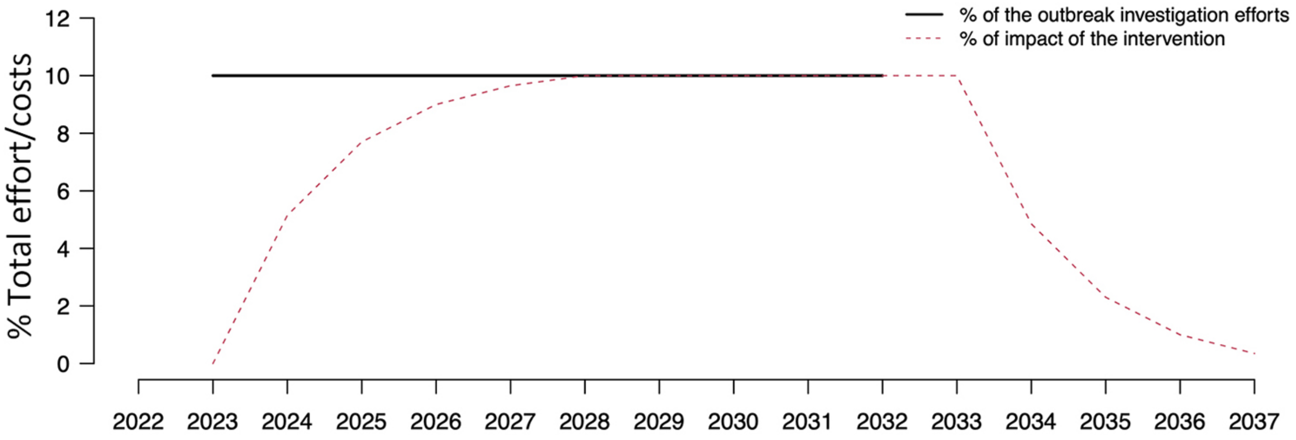

Appendix Figure 1. Analytic time horizon. The outbreak investigations are implemented over a 10-year period between 2023–2032, with efforts and costs distributed equally (i.e., 10% each year, shown in a solid black line). The impact of the intervention is evaluated over a 15-year period between 2023–2037, accounting for impacts that occur up to a 5-year period post-intervention. The percentage of the overall impact that is expected to occur during the 15-year period is shown by the dashed red line.

Sensitivity analyses of TB cases averted

As shown in Appendix Figure 2, the factors that were most influential to the estimated epidemiologic impact of outbreak investigation activities included (i) the number of contacts investigated per case during outbreak investigation; (ii) the percentage of LTBI cases reactivating within 5 years; and (iii) the reproductive number, R_0. The estimated number of TB cases averted due to outbreak investigation was less sensitive to variation in other model parameters.

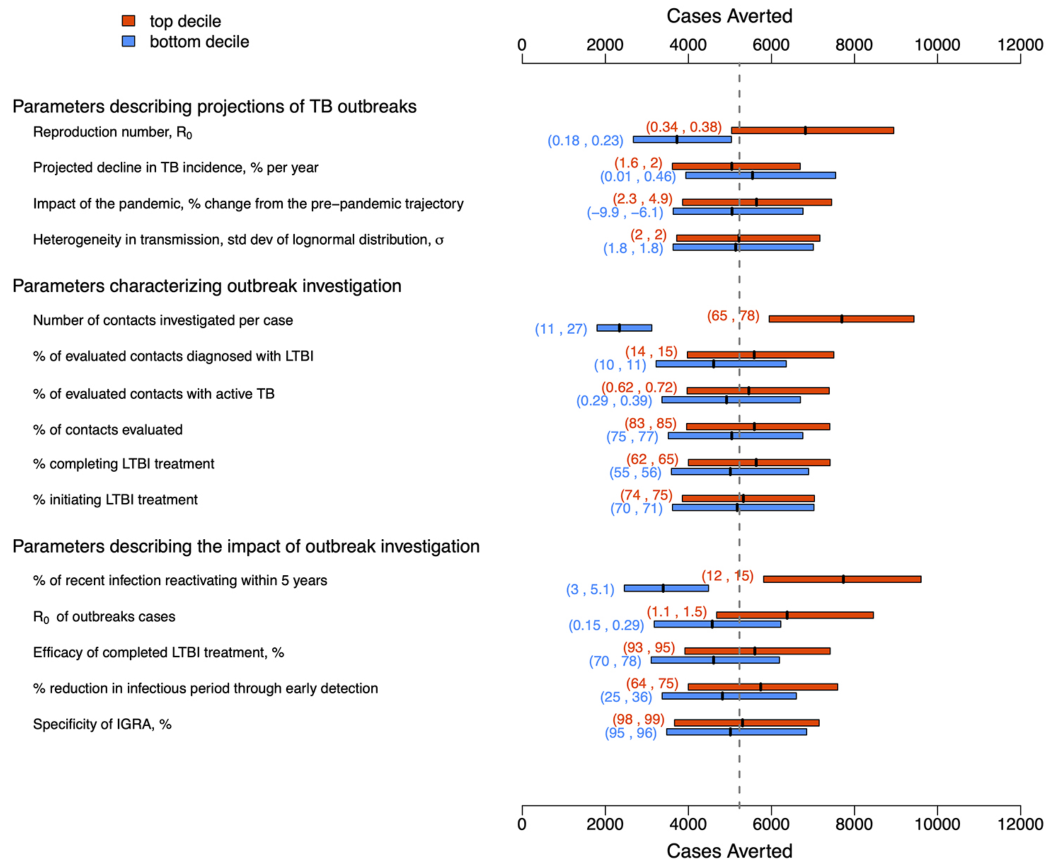

Appendix Figure 2. Multivariate sensitivity analysis of the model parameters on the epidemiologic impact of TB outbreak investigation. This graph illustrates the sensitivity of the epidemiologic impact of TB outbreak investigation in the U.S. (in the number of TB cases averted during 2023–2037) to the values of individual model parameters. Each pair of boxplots shows variation in the outcome when the analysis was limited to either simulations in which the value of the parameter of interest was in the top (red) or bottom (blue) decile of its values across all simulations. The edges of each box represent the lower and upper interquartile range, and the band in the middle represents the mean. The vertical dotted line shows the mean across all simulations (5,560 cases averted).

Estimating cost-effectiveness with alternative parameter distributions.

In our main analysis, we sampled our model parameters from triangular distributions. In this supplementary analysis, we sampled our model parameters from the following distributions: (i) PERT distributions, where the mode of the distribution was taken to be the point estimate, and the minimum and maximum of the distribution were, respectively, the lower and the upper values shown in Table 1 in the main text or (ii) a mixture of PERT and gamma distributions, where, in addition to (i), cost parameters were sampled using gamma distributions. For each parameter, the gamma distribution was

chosen so that the mean was equal to the point estimate, and the 95% were roughly equal to the lower and upper values shown in Table 1 in the main text.

As shown in Appendix Figure 3, both the epidemiologic impact and the cost-effectiveness estimates did not vary substantially between the three parameter distributions we compared. Specifically, the estimated number of TB cases averted was 5,560 (1,720–11,400), 5,400 (2,030 – 10,500), and 5,460 (2090 –10700), respectively, for triangular, PERT, and mixed distributions; and the cost per QALY gained (in 2022 U.S.$) was $27,800 (4,580–68,700), $32,100 (8,240–72,800) and $21,600 (613–55,500), respectively, for triangular, PERT and mixed distributions.

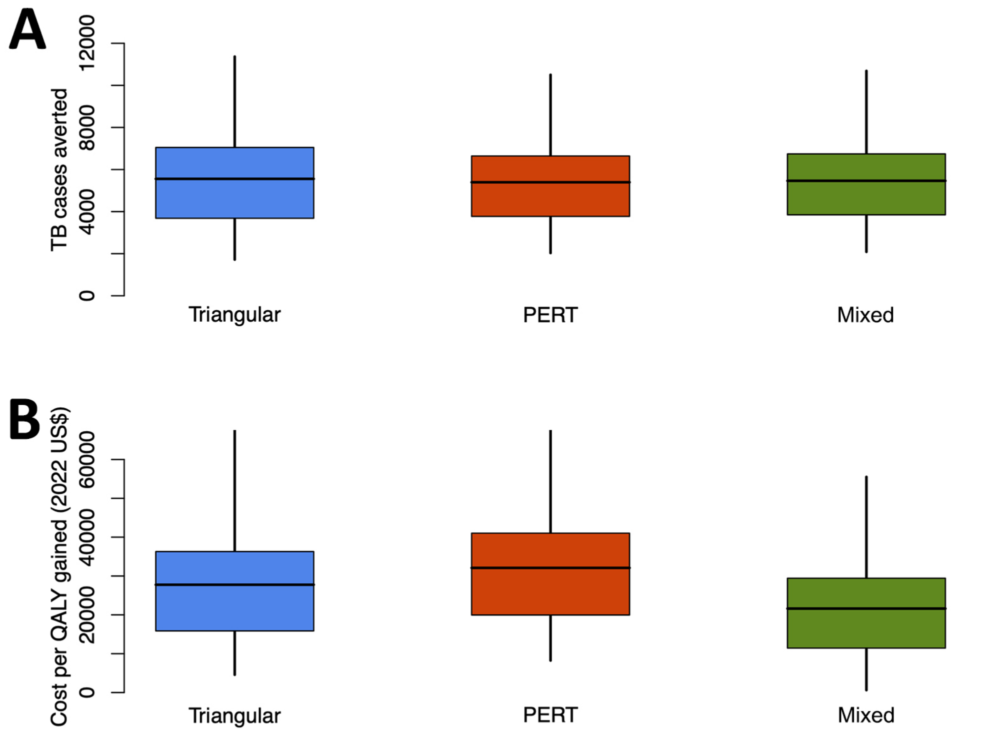

Appendix Figure 3. Comparing model estimates when parameters were sampled from Triangular, PERT, and mixed distributions. Shown are the epidemiologic impact (panel A, TB cases averted during 2023–2037) and cost-effectiveness (panel B, Cost per QALY gained, 2022 U.S.$) of the outbreak investigation in the United States. Shown in blue box plots are the results when the model parameters were sampled from Triangular distributions, in red box plots are the results when the model parameters were sampled from PERT distributions, and in green box plots are the results when the model parameters were sampled from a mixture of PERT and gamma distributions.

References

1. Arias E, Xu J. United States Life Tables, 2019. National Vital Statistics Report, Vol 70 No 19 [cited 2023 Mar 31]. https://www.cdc.gov/nchs/data/nvsr/nvsr70/nvsr70-19.pdf

2. Bauer M, Ahmed S, Benedetti A, Greenaway C, Lalli M, Leavens A, et al. Health-related quality of life and tuberculosis: a longitudinal cohort study. Health Qual Life Outcomes. 2015;13:65. PubMed https://doi.org/10.1186/s12955-015-0250-4

3. Dale KD, Abayawardana MJ, McBryde ES, Trauer JM, Carvalho N. Modeling the cost-effectiveness of latent tuberculosis screening and treatment strategies in recent migrants to a low-incidence setting. Am J Epidemiol. 2022;191:255–70. PubMed https://doi.org/10.1093/aje/kwab150

4. Tasillo A, Salomon JA, Trikalinos TA, Horsburgh CR Jr, Marks SM, Linas BP. Cost-effectiveness of testing and treatment for latent tuberculosis infection in residents born outside the United States with and without medical comorbidities in a simulation model. JAMA Intern Med. 2017;177:1755–64. PubMed https://doi.org/10.1001/jamainternmed.2017.3941

5. Sassi F. Calculating QALYs, comparing QALY and DALY calculations. Health Policy Plan. 2006;21:402–8. PubMed https://doi.org/10.1093/heapol/czl018

6. Jo Y, Shrestha S, Gomes I, Marks S, Hill A, Asay G, et al. Model-based cost-effectiveness of state-level latent tuberculosis interventions in California, Florida, New York, and Texas. Clin Infect Dis. 2021;73:e3476–82. PubMed https://doi.org/10.1093/cid/ciaa857

7. Guo N, Marra CA, Marra F, Moadebi S, Elwood RK, Fitzgerald JM. Health state utilities in latent and active tuberculosis. Value Health. 2008;11:1154–61. PubMed https://doi.org/10.1111/j.1524-4733.2008.00355.x

8. Salomon JA, Haagsma JA, Davis A, de Noordhout CM, Polinder S, Havelaar AH, et al. Disability weights for the global burden of disease 2013 study. Lancet Glob Health. 2015;3:e712–23. PubMed https://doi.org/10.1016/S2214-109X(15)00069-8

9. Mitruka K, Oeltmann JE, Ijaz K, Haddad MB. Tuberculosis outbreak investigations in the United States, 2002–2008. Emerg Infect Dis. 2011;17:425–31. PubMed https://doi.org/10.3201/eid1703.101550

10. Mindra G, Wortham JM, Haddad MB, Powell KM. Tuberculosis outbreaks in the United States, 2009–2015. Public Health Rep. 2017;132:157–63. PubMed https://doi.org/10.1177/0033354916688270

11. Centers for Disease Control and Prevention (CDC). 2020 Contact Investigations Report (ARPE Data) [cited 2023 Mar 31]. https://www.cdc.gov/tb/programs/evaluation/arpe-data.htm

12. Shrestha S, Winglee K, Hill AN, Shaw T, Smith JP, Kammerer JS, et al. Model-based analysis of tuberculosis genotype clusters in the United States reveals high degree of heterogeneity in transmission and state-level differences across California, Florida, New York, and Texas. Clin Infect Dis. 2022;75:1433–41. PubMed https://doi.org/10.1093/cid/ciac121

13. Kammerer JS, Shang N, Althomsons SP, Haddad MB, Grant J, Navin TR. Using statistical methods and genotyping to detect tuberculosis outbreaks. Int J Health Geogr. 2013;12:15. PubMed https://doi.org/10.1186/1476-072X-12-15

14. Behr MA, Edelstein PH, Ramakrishnan L. Revisiting the timetable of tuberculosis. BMJ. 2018;362:k2738. PubMed https://doi.org/10.1136/bmj.k2738

15. Menzies NA, Swartwood N, Testa C, Malyuta Y, Hill AN, Marks SM, et al. Time since infection and risks of future disease for individuals with *Mycobacterium tuberculosis* infection in the United States. Epidemiology. 2021;32:70–8 PubMed https://doi.org/10.1097/EDE.0000000000001271
